# *Aloe vera* Flowers, a Byproduct with Great Potential and Wide Application, Depending on Maturity Stage

**DOI:** 10.3390/foods9111542

**Published:** 2020-10-26

**Authors:** Ascensión Martínez-Sánchez, María Elena López-Cañavate, Josefa Guirao-Martínez, María José Roca, Encarna Aguayo

**Affiliations:** 1 Food Quality and Health Group, Institute of Plant Biotechnology, Universidad Politécnica de Cartagena (UPCT), Campus Muralla del Mar, 30202 Cartagena, Spain; ascension.martinez@upct.es (A.M.-S.); elenicalc@gmail.com (M.E.L.-C.); nafi1993-24@hotmail.com (J.G.-M.); 2 Postharvest and Refrigeration Group, Escuela Técnica Superior de Ingeniería Agronómica (ETSIA), UPCT, Paseo Alfonso XIII, 48, 30203 Cartagena, Spain; 3 Servicio de Apoyo a la Investigación Tecnológica (Support Service for Technological Research), UPCT, Campus Muralla del Mar, 30202 Cartagena, Spain; mjose.roca@upct.es

**Keywords:** omega-3, trigonelline, residue, sábila, crassulaceae, added-value products

## Abstract

Flowers of *Aloe vera* are a byproduct providing a valuable source of bioactive compounds with different functions for health benefits. The characterization in amino acids, organic acids, sugars, trigonelline, volatiles compounds, fatty acids, total phenolic, carotenoids, vitamin C content, and antioxidant capacity of Aloe flowers (*Aloe barbadensis* Miller) has been studied at three maturity stages (I: immature; II: mature; III: mature, with flowers buds opened). Immature flowers presented the highest content in phenyl alanine, tyrosine, citric acid, trigonelline, carotenoids, retinol activity equivalent, vitamin C, and total phenolic and antioxidant capacity. As the flower develops, the content of these compounds decreases. *Aloe vera* flowers presented an important content in fatty acids, and the principal concentration was identified in polyunsaturated unsaturated fatty acids (PUFAs) as α-linolenic acid, and linoleic acid, with a ratio close to one. The main saturated fatty acid was palmitic acid, followed by stearic acid. Maturity stage III showed the lowest fatty acid content. The bioactive compounds found in *Aloe vera* flowers have potential applications in the cosmetic, pharmaceutical, nutraceutical, and food industries. Depending on the compound of interest, it could be worthwhile harvesting flowers at maturity stage I, thereby reducing the energy consumption of flowers from the plant and thus favoring plant development. This is an example of a circular economy for *Aloe vera* producers, generating economic and business opportunities and thus providing environmental and social benefits.

## 1. Introduction

*Aloe vera* is a plant that has been used for thousands of years for its medicinal properties to treat different human diseases and disorders [1]. Several studies have reported different bioactive compounds such as amino acids, anthraquinones, enzymes, sugars, polyphenols, minerals, and vitamins (A, B, C, and E) in *Aloe vera* leaves and gel [2,3,4,5]. These bioactive compounds provide healthy properties, such as anti-ulcer, anti-hypercholesterolemic, antioxidant, antibacterial activity, antiviral activity, antifungal activity, anti-acne, nutraceutical, humectant, skin protection against UV-A and UV-B, wound healing properties, the prevention of type II diabetes and cancer, cardiovascular diseases, and the generation of antibodies [6,7]. Recently, the use of *Aloe vera* has been expanded to the food sector with the production of beverages and food supplements of *Aloe vera*–based products, as well as edible fruit coatings for grapes, cherries, or papaya. It is also a source of bioactive natural compounds in the pharmaceutical and cosmetics industries due to its antioxidant, humectant, antibacterial, and antifungal activity [8,9].

Leaves are the most used part of the plant, with bioactive acetylated glucomannan and anthraquinone glycosides being obtained from the Aloe gel, while the latex contains too high an anthraquinoids content (aloin A and B); an excessive consumption of these compounds presented in aloe juice can have toxic effects, and therefore, its consumption must be limited [10]. Recently, some studies have reported the antioxidant properties [11,12] or carotenoids content, composition of phenolic compounds, and fatty acid profile of *Aloe vera* flowers [13,14]. In some countries, the flowers have become a new crop with great popularity in the culinary field, as they are rich in nutrients and bioactive compounds [15,16,17,18]. The *Aloe vera* plant has an inflorescence in the form of a simple or compound cluster, capable of containing 100–200 hermaphroditic flowers, which are arranged helically on an erect leaking of approximately 90–100 cm in length that sprouts through the center of the plant. The maturity state of these yellow-colored tubular flowers is gradual from the bottom to the top, which points to quantitative and qualitative differences of the bioactive compounds present in the flowers. With certain exceptions, *Aloe vera* flowers are considered a residue without application in any industry. Farmers usually cut the inflorescence from its base to maintain the vigor of the aloe leaves [19]. However, Quispe et al. [3] described an antioxidant activity four to eight times greater in the flowers than in the gel. Furthermore, some bioactive compounds such as gentisic acid with anti-inflammatory, antirheumatic, and antioxidant properties have been detected mainly in the flowers (101 mg/100 g freeze-dried) rather than in the leaf skin (6 mg/100 g freeze-dried) [16]. Aloe flowers presented an important content of apigenin glycoside derivatives (4.48 mg/g), and no anthraquinone glycosides have been detected in that part of the plant [13]. Additionally, the important antimicrobial activity of *Aloe vera* flower against the multidrug-resistant *P. aeruginosa* (MIC = 0.025 mg/mL and MBC = 0.05 mg/mL) and the powerful antifungal activity against *A. flavus*, *A. niger*, *P. funiculosum*, and *C. albicans*, higher than that of ketoconazole, should be highlighted [13]. Therefore, Aloe flowers could be considered as a valuable byproduct for the food, cosmetic, or pharmaceutical industries. However, the Aloe flower is not a plant material that has been studied to the same extent as the leaves. The optimal time to harvest aloe leaves is reportedly after three years of the plant’s growth, because then it has the highest content of bioactive compounds [12], but the best maturity stage to harvest Aloe flowers remains unknown.

The main objective of this study was to determine the bioactive potential of *Aloe vera* (*Aloe barbadensis* Miller) flowers and the characterization of three different development stages of flowers: stage I (immature), stage II (mature, but flower buds still closed), and stage III (mature, with flower buds opened). Carbohydrates, organic acids, amino acids, volatile profile, fatty acids content, carotenoids content, retinol activity equivalent, and other bioactive compounds were studied in the *Aloe vera* flowers.

## 2. Materials and Methods 

### 2.1. Plant Material

**Figure 1:**
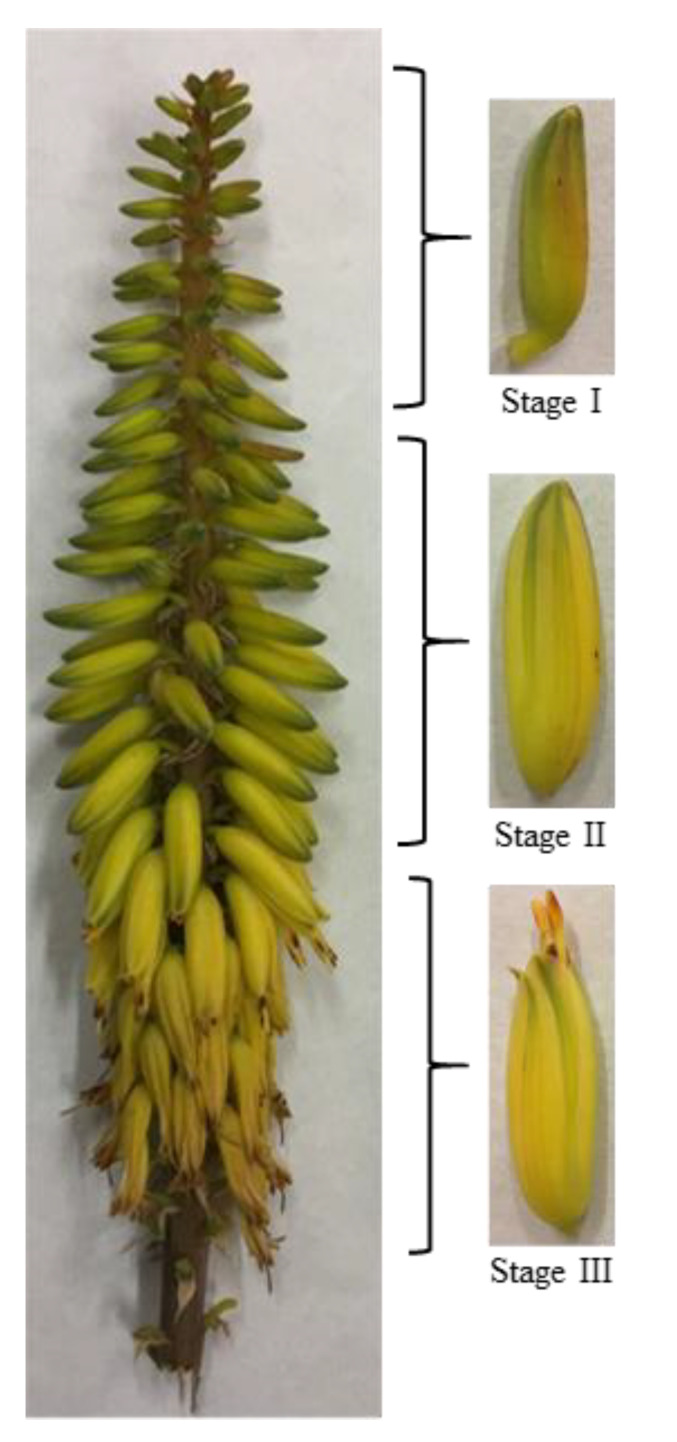
Inflorescence of aloe plant and classification of *Aloe vera* flowers according to their state of development: stage I (immature flowers shorter than 2 cm), stage II (mature, but flower buds still closed, flowers between 2–4 cm), and stage III (mature, with flowers buds opened, more than 4 cm).

*Aloe vera* (*Aloe barbadensis* Miller) plants were cultivated under commercial organic grown conditions in Águilas (Murcia, Spain) under Mediterranean climate, with low rainfall (~ 260 mm per year), by the company Ecoagrícola El Talayón S.L. The plant material consisted of four-year-old *Aloe vera* plants, 6000 plants per hectare, with 1 m × 1.2 m plant spacing. Plants were drip irrigated (1200 m^3^/ha year) using a drip irrigation line for each row, with two emitters per plant, each one with a flow rate of 2 L/h. In May, for this Mediterranean area, the racemes from the inflorescence from 50 *Aloe vera* plants were harvested manually, plant by plant, using hand-held knives and transported by car (80 km) under refrigerated conditions in polyspan boxes to the UPCT laboratory (Cartagena, Spain). Racemes were stored at 10 °C until the following morning, and then the flowers were manually separated from the raceme. The flowers were classified according to their length at three different states of development: stage I (immature) 2-cm flowers; stage II (mature, but flower buds still closed) flowers between 2–4 cm long; and stage III (mature, with flower buds opened) 4-cm flowers (Figure 1). The flowers were frozen in liquid nitrogen, ground, and maintained at −80 °C until the analysis for vitamin C, total phenolic compounds, and antioxidant capacity. For the analysis of primary metabolites, volatile profile, fatty acid profile, carotenoids, and retinol activity equivalent (RAE), the powder of frozen flowers was freeze-dried (Telstar Lyoquest 85 plus, Eco, Barcelona, Spain) for 48 h and maintained in sealed plastic bags in a box with moisture absorbers until characterization. The ratio between fresh and dry weight was of 1:0.17 for flowers in stage I (83% moisture), 1:0.14 for stage II (86% moisture), and 1:0.15 (g/g) for flowers in stage III (85% moisture).

### 2.2. Primary Metabolites Analyses

The determination of primary metabolites (amino acids, free sugar composition, organic acids, nucleoside derivatives and choline, trigonelline and ethanol) was carried out by ^1^H nuclear magnetic resonance (NMR), (AVANCE III HD 500 MHz, CryoProbe Prodigy BBO, Bruker, Billerica, MA, USA). Freeze-dried *Aloe vera* powder flower samples (50 mg) were extracted with 0.5 mL of D_2_O (deuterated water). The internal standard, trimethylsilylpropanoic acid (TSP) 2.9 mM was added. Samples were vortexed for 5 min, transferred to NMR spectrometry tubes, and analyzed according to Biais et al. [20]. All ^1^H NMR spectra were recorded at 298 K on a Bruker AVIII HD 500 NMR spectrometer (500.13 MHz for ^1^H) equipped with a 5 mm CPP BBO cryogenic probe (Bruker Biospin, Germany). ^1^H spectra were referenced to TSP signal (δ = 0.00 ppm). Concerning to NMR measurements, for each sample, 32 scans were recorded with the following parameters: 0.126 Hz/point, pulse width (PW) = 4.0 μs (30°), and relaxation delay (RD) = 2.0 s. FIDs were Fourier transformed with LB = 0.5 HZ, GB = 0, and PC = 1.0. For quantitative analysis, peak integral was used. The spectra were referenced to trimethyl silane propionic acid sodium salt (TSP) at 0.00 ppm for aqueous phase. TSP, 0.01%, *w/v* were used for internal standard. D_2_O was used as the internal lock. The whole peak intensities in every 0.02 ppm in ^1^H NMR spectra in the range of δ 0.30–12.5 were used as variables. ^1^H NMR spectra were manually corrected for phase and baseline distortions using TOPSPIN (v3.2, Bruker Biospin). Peak-fitting on the resulting spectra was done using a computer algorithm associated with Chenomx NMR Suite 8.1 software (Billerica, Massachusetts, US) to generate concentrations (in µM) of metabolites were determined using the 500 MHz library from the mentioned software, which compares the integral of a known reference signal (TSP) with signals derived from a library of compounds containing chemical shifts and peak multiplicities. The region δ = 4.67–5.15 was discarded to eliminate the effects of imperfect water presaturation. The spectral areas of all buckets were normalized to the weight of extracts employed for measurements. The intensities 23 selected ^1^H resonances due polar metabolites were measured with respect to the intensity of TSP signal used as internal standard with a concentration of 0.29 mM. The samples were evaluated by triplicate for each maturity stage and the results were expressed in mg/g per dry weight (d.w.).

### 2.3. Volatile Profile

Volatile compounds were only identified (not quantified) by solid phase microextraction (SPME) using a divinylbenzene/carboxenide/polydimethylsiloxane fiber (DVD/CAR/PDMS) (Supelco, Bellefonte, PA, USA) [21] using a gas chromatograph (GC) Agilent 6890 coupled 5975 Inert Mass Selective detector (GC-MS, Agilent Technologies, Palo Alto, CA, USA). Before analysis, the SPME fiber was conditioned for 60 min at 270 °C, and just before the analysis, it was reconditioned for 5 min at 270 °C in the GC injection port. Freeze-dried *Aloe vera* flower samples (0.4 g) were weighed into 10 mL vials and immediately sealed with a silicone-Teflon screw cap and placed on a 75 °C heating plate for 15 min. After this, the SPME fiber was introduced into the vial for 10 min at 75 °C to absorb the headspace volatiles of samples. Thermal desorption was carried out by transferring the SPME fiber to the GC injection port for 3 min at 250 °C. Volatile compounds were separated on a HP-5MS (30 m × 0.25 mm × 0.25 μm film thickness, 5%-diphenyl-95%-phenyl methyl siloxane) capillary column (Agilent) with helium as the carrier gas (1.3 mL/min) and constant pressure mode. Chromatographic separation was conducted as follows: initial oven temperature was 50 °C, then increased up to 80 °C (5 °C/min gradient), then up to 100 °C (10 °C/min gradient), and finally increased to 185 °C (5 °C/min gradient). Injections were conducted in split mode (1:30) and using 250 °C injector temperature. Mass spectra, using a 70 eV electron impact ionization source, were scanned in the range m/z 40–400 amu, and the identified peaks were carried out in full scan mode. The source and quadrupole temperatures were set at 230 °C and 150 °C. 

GC/MSD ChemStation Software (G1701EA D.02.00.275, Agilent Technologies) was used to integrate and identify the peaks. Individual peak identification was based on the comparison of their mass spectra with the reference mass spectra of National Institute of Standards and Technology (NIST 11) and Wiley 9th Ed. libraries. Volatile organic compounds (VOCs) were qualitatively expressed as percentages of the total area counts recorded in each chromatogram and qualitatively identified considering match qualities above 80%.

### 2.4. Fatty Acid Profile

The content of fatty acids was determined in freeze-dried powder flower samples according to Smooker et al. [22], with some modifications. Powder (0.4 g) was mixed with 1 mL of hexane and 5 μL of internal standard 0.2 M, nonadecanoic acid (C19:0) resuspended in methanol:toluene:2,2-dimethoxypropane:sulfuric acid (33:14:2:1). The mixture was incubated in a water bath for 1 h at 80 °C, then samples were cooled at room temperature and the organic phase was separated, which was injected into the gas chromatograph (GC) (Agilent 6890 coupled 5975 Inert Mass Selective detector (GC-MS, Agilent Technologies, Palo Alto, CA, USA) equipped with a DB-23 column (60 m × 0.25 mm × 0, 25 μm; Supelco) and using nitrogen as carrier gas and a flame ionization detector (FID) at 250 °C. Fatty acid compounds were identified and quantified by comparing with the commercial mix of FAME standards (37 components mix, Supelco Co., Bellefonte, PA, USA). The results were expressed in mg/100 g d.w. 

### 2.5. Carotenoids and RAE

Freeze-dried powder of *Aloe vera* flower samples (0.04 g) was extracted in 7 mL of ethanol:hexane (4:3) [23] with some modifications. Samples were stirred for 60 min at 200 rpm at 4 °C on an orbital shaker (SSL1, STUART, Staffordshire, UK) under darkness conditions. After this, distilled water (1 mL) was added to each sample and stirred for 5 min at 200 rpm at 4 °C. Finally, carotenoids content (α-carotene, β-carotene, β-cryptoxanthin, zeaxanthin and lycopene) from the organic phase was determined by UV- visible spectrophotometry (UV-1603 Schimadzu, Tokyo, Japan) at 444, 450, 451, 451, and 472 nm, respectively, and quantified using the extinction coefficients (ƐmM^−1^ cm^−1^) in hexane: 150.3, 137.4, 136, 141.1, and 185.3 for α-carotene, β-carotene, β- cryptoxanthin, zeaxanthin, and lycopene, respectively, according to Hart and Scott [24]. The results were expressed in mg/100 g d.w. Additionally, Retinol Activity Equivalents (RAE) were calculated according to Ordóñez-Santos et al. [23] as the mg of β-carotene divided by 12 plus the mg of other provitamins A (α-carotene and β-cryptoxanthin) divided by 24, with the results being expressed in mg RAE/100 g d.w.

### 2.6. Vitamin C

The content of total vitamin C was calculated as the sum of ascorbic acid (AA) and dehydroascorbic acid (DHAA), according to the method described by Martínez-Sánchez and Aguayo [25]. Frozen fresh flower samples (5 g) were homogenized in an ultraturrax (Ultra-Turrax IKA-T 18 Basic, Darmstadt, Germany) for 30 s in 10 mL of the extraction medium (MeOH/H_2_O (5:95) plus citric acid (21 g L^−1^) with EDTA (0.5 g L^−1^). Standard solutions, column conditioning, mobile phase, flow rate, wavelengths and derivatization procedures were previously reported by Martínez-Sánchez et al. [26]. Results were expressed in mg/100 g of f.w. (fresh weight).

### 2.7. Total Phenolic Compounds

The total phenolic compounds (TPC) were determined by spectrophotometry according to Singleton and Rossi [27] with some modifications. Frozen fresh flower powder of *Aloe vera* (0.3 g) was homogenized for 30 s by Ultra-Turrax (T-25, Ika-Labortechnik, Staufen, Germany) with 10 mL of methanol:water (8:2) containing of sodium fluoride (NaF) 4 mM. The extracts were centrifugated at 12,500× *g* (Sigma 12,002 rotor, Sigma, Saint Louis, MO, USA) for 8 min at 4 °C, and supernatants were used to determine the TPC by the reduction of Folin-Ciocalteau reagent [25]. The absorbance was measured at 750 nm at 25 °C in a Tecan Infinite^®^ 200 micro plate reader (Grödig, Austria), and the results were expressed as mg of gallic acid equivalents (GAE) per 100 g of f.w.

### 2.8. Total Antioxidant Capacity

The total antioxidant capacity (CAT) was determined using three analytical methods according the ability to reduce free DPPH^•^ and ABTS^•+^ radicals or to reduce ferric iron to ferrous, the FRAP assay. Different extracts were used for DPPH, ABTS, and FRAP. DPPH assay was determined from TPC extract following the Brand-Williams et al. [28] method with some modifications, the changes of absorbance at 515 nm were determined after 1 h of reaction at room temperature in darkness conditions. The extracts from vitamin C, without filtering through a C18 Sep-Pak cartridge, were used to determine the ABTS and FRAP assays [25]. In these analyses, the changes in absorbance were determined after 40 min of reaction at room temperature at 414 nm and 593 nm for the ABTS and FRAP assays, respectively. Trolox was used as the standard and the antioxidant activity was expressed as mg of trolox equivalent antioxidant capacity (TEAC) per 100 g of f.w.

### 2.9. Statistical Analysis

For each determination, three replicates per maturity stage of *Aloe vera* flowers were evaluated, and an analysis of variance (ANOVA) was performed to compare different maturity stages at a significance level of *p* ≤ 0.05 using PASW Statistics 25 for Windows (SPSS Inc., Chicago, IL, USA) and, when significant differences were observed, the Tukey’s honestly significant difference (HSD) test was applied.

## 3. Results and Discussion

### 3.1. Primary Metabolites

#### 3.1.1. Amino Acids

Table 1 shows the main amino acids found in *Aloe vera* flowers. Four of the nine amino acids identified were essential (phenylalanine, threonine, valine, and isoleucine). Glutamine was the major amino acid present in *Aloe vera* flowers (0.613 ± 0.027 to 0.748 ± 0.076 mg/g d.w.). The different maturity stages affected the aspartate, phenylalanine, and tyrosine content; flowers in maturity stage I presented the highest concentration. Although there are no previous studies regarding the variation of amino acid content with *Aloe vera* flower development, the maturity stage of flowers is probably influenced in different metabolic pathways that these amino acids could be involved in. The increase in free amino acid content during the immature stages of flowers could be due to a de novo synthesis, whilst in the mature stages (II and III), amino acids are probably involved in the catalytic pathway. Phenylalanine and tyrosine belong to shikimic acid family, and both amino acids are the precursors use in the biosynthesis of polyphenolic compounds such as cinnamic acids, tannins, and flavonoids. Therefore, these amino acids could be involved in the synthesis of chemical compounds linked to the color and aromatic volatile compounds of Aloe flowers [29]. Joshi et al. [30] found that the amount of protein per unit dry weight was highest in the youngest stage and lowest in the later stages of tea flower development. According to these authors, soluble protein concentration decreased from bud to stage 4 of tea flower development, which suggested an increased protein synthesis during the rapid bud development phase and decline thereafter in the flower expansion stages. The rest of the amino acids were not affected by the maturity state of *Aloe vera* flower. Sotelo et al. [15] identified in *Aloe vera* (“sábila”) flowers the same amino acids plus proline, leucine, serine, arginine, glycine, lysine, histidine, cysteine, methionine, and tryptophan. Those researchers did not find gamma γ-aminobutyric acid (GABA), a non-proteinogenic amino acid. However, glutamine was also the main amino acid (15.09 ± 0.44 mg/g d.w). Mulu et al. [31] reported that *Aloe vera* gel (whole leaf) provided 20 of 22 required amino acids and 7 of 8 essential ones. In tea flowers, the total amount of free amino acids was 8.09 mg/g d.w. [32], much higher than the 1.76 to 1.95 mg/g quantified in *Aloe vera* flowers. The presence and concentration of amino acids in *Aloe vera* flowers has nutritional and cosmetic interest since most of them have beneficial effects on the skin, softening and moisturizing the cells [6], and they are the building blocks of proteins, which manufacture and repair muscle tissue [31].

#### 3.1.2. Free Sugar Composition

Glucose (7.57 ± 0.53 to 42.78 ± 7.24 mg/g dw), fructose (8.14 ± 0.25 to 28.57 ± 0.88 mg/g dw) and sucrose (3.12 ± 0.83 to 11.07 ± 0.92 mg/g dw) were the main carbohydrates presented in *Aloe vera* flowers (Table 1). Trehalose was also identified but at a low concentration (0.34 ± 0.02 to 0.10 ± 0.00 mg/g d.w.). Both monosaccharides and trehalose increased with the development of the flowers, whilst sucrose decreased with the advanced maturity state. This same trend was described in azalea flowers (*Rhododendron simsii hybrids*), glucose and fructose concentrations, and invertase activity increased in azalea petals during flowering, while sucrose decreased [33]. *Aloe vera* flowers presented a high relative concentration of acetylated polysaccharide in the maturity stages I and II and lower in stage III (data not shown). Chang et al. [34] identified glucose, galactose and mannose as major sugars and xylose and rhamnose in lower concentration in *Aloe vera* flowers. Other researchers have reported that in *Aloe vera* gel, the carbohydrate fraction is the higher proportion (0.25%) of the total gel composition (25–50% solid component of the fraction). Monosaccharides include mannose, free glucose, fructose, and galactose [35]. Bozzi et al. [4] quantified glucose (11.85 g/100 g d.w.) and fructose (5.3 g/100 g d.w.) as the predominant free sugars and also a lower quantity of sucrose (0.16 g/100 g d.w.) and mannose (0.08 g/100 g d.w.) in fresh frozen dice of *Aloe vera* gel. Fernandes et al. [36] evaluated the composition of the four edible flower species (borage, camellia, centaurea and pansies). The free sugars identified were glucose, fructose, and sucrose, varying between 17.4 mg/g to 131 mg/g dw, 27.1 mg/g to 166 mg/g d.w., and 4.9 mg/g and 38.6 mg/g dw, respectively. Our data are in the range for free sugar composition in flowers. In *Aloe vera*, the monosaccharides (glucose and fructose) and polysaccharides (glucomannans) have been linked to anti-viral properties or to the immune modulating activity of acemannan [6]; for that reason, *Aloe vera* flowers could have pharmaceutical potential in this field.

#### 3.1.3. Organic Acids 

Two organic acids, citric and malic acid, presented higher concentrations than acetic, formic, and fumaric acids (Table 1). The content of all the organic acids identified was affected by the flower maturity stage, except for fumaric acid. Citric acid decreased with flowers that were open but acetic and formic acid increased the level. Those acids usually have an impact on the color and flavor of fruits and vegetables. Fernandes et al. [36] identified eight organic acids in almost all edible flowers species, with malic acid (18.4–49.2 mg/g dw) being the major organic acid. Bozzi et al. [4] reported malic acid as the only organic acid contained in fresh *Aloe vera* gel. According to those researchers, malic acid is the storage of carbon dioxide for plant photosynthesis and citrate is involved in the main processes of energy and biosynthetic metabolism. However, Flores-López et al. [37] reported the profile of organic acid in *Aloe vera* gel, noting the presence of a high concentration of malic acid (18.17%), followed by acetic (3.65%) and citric acid (0.36%). Malic acid is a quality parameter in the processing of *Aloe vera*. This acid is formed in *Aloe vera* gel as a result of typical crassulacean acid metabolism, usually in the range of 11.10% and 40.40% [38]. The presence of other organic acids as acetic, formic, fumaric, succinic, or lactic acid in *Aloe vera* gel could suggest microbial and enzymatic degradation [4]. In our case, acetic and formic acid increased slightly in stage III, which could indicate the first steps of the end of the flowers’ shelf-life. The major organic acids found in *Aloe vera* flowers as malic and citric acid have been related to specific key roles in human health. For example, citric acid is a crystal thickener in bones [39], and malic acid has an antimicrobial action against specific pathogenic bacteria [40] and protective effects on myocardial ischemia/reperfusion injury [41].

Depending on the nucleoside, adenosine monophosphate (AMP) and adenosine derivatives were found (Table 1). These compounds participate in various metabolic pathways such as the Krebs cycle, among others. No significant differences were observed in the adenosine content among the different maturity stages, although AMP decreased gradually throughout flower development, thus suggesting that senescence is closely related with energy. On the other hand, other metabolites such as choline, ethanol, and trigonelline showed a decrease as the flowers developed. Choline is a component of phosphatidylcholine, one of the main molecules of the lipid bilayer that makes up cellular membranes. Other authors have found more of this phospholipid in immature fruits than in mature fruits [42], so the higher concentration of choline in immature flowers could be related to greater stability of the cell membrane [43] and with their greater firmness. The lower concentration of choline in mature flowers could be explained by the degradation of phosphatidylcholine associated with a loss of the cell membrane properties associated with senescence [44].

Trigonelline (N-methylnicotinate) is a specific plant metabolite, an alkaloid mostly reported from the Fabaceae family, and is biosynthesized from nicotinate. Several authors have reported the positive properties of trigonelline, such as anti-diabetic, antioxidant, anti-inflammatory, hypocholesterolemic, and neuroprotective effects [45], and it can be used to prevent skin photo-damage [46]. Mathur and Kamal [47] also quantified trigonelline in *Moringa oleifera* flowers (1.60 mg/g f.w.). Nowadays, coffee is the main source of the alkaloid trigonelline, at around 5–17 mg/g coffee [48]. The present study is the first report of the presence of trigonelline in *Aloe vera* flowers. Regarding the ethanol concentration, it decreased with the flower maturity state (Table 1), suggesting its role during the senescence process. For example, postharvest ethanol application can prolong the longevity of cut flowers [49] reducing the internal ethylene concentration.

### 3.2. Volatile Compounds

**Figure 2:**
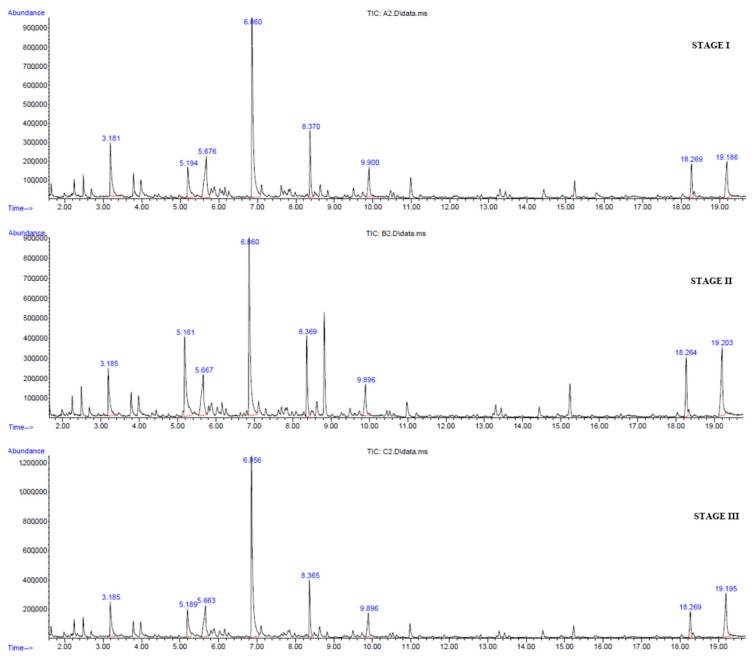
Profile of volatile compounds of the *Aloe vera* flowers at maturity stages I, II, and III. 1: 2-hexenaldehyde; 2: benzaldehyde; 3: 1-hexanoic acid; 4: benzyl alcohol; 5: 1-nonanal; 6: octanoic acid; 7: dodecanoic acid methyl ester; 8: dodecanoic acid.

The chromatographic profiles are similar for the three states under study (Figure 2). Table 2 shows the compounds with quality match between the non-target compound and the library compound to be higher than 80%. Moreover, it includes the percentage contribution to the total area of peaks found of volatile compounds of the *Aloe vera* flower in the three maturity stages in this study (stages I, II, and III). Among the VOCs found, those with the highest relative percentages were benzyl alcohol, 1-nonanal, lauric acid, 2-hexenaldehyde, caproic acid, benzaldehyde, dodecanoic acid methyl ester, and caprylic acid. All of them were present in the three stages of the *Aloe vera* flower, with benzyl alcohol clearly standing out as the majority component of the aromatic profile, presenting a relative percentage above 20% (stages I and III). Benzyl alcohol was one of the main compounds of the jasmine flower and is involved in the relief of headaches, coughs, chronic diseases, etc. [50]. This compound was also identified in yellow tea in the study by Guo et al. [51], although in that case, it was not the majority compound. On the other hand, compounds such as benzaldehyde are used as flavoring in foods and cosmetics and a precursor to pharmaceutical compounds [52]. Additionally, Mekni et al. [53] observed a lower minimum inhibitory concentrations (MIC) for *Staphylococcus aureus* and *Enterococcus faecalis* in *Tunisian Punica granatum* L. flower cultivars with higher volatile compounds including alcohols (r = 0.96, *p* < 0.05 for *E. faecalis* only), aldehydes (r = 0.67 and r = 0.99; *p* < 0.05, respectively), unsaturated hydrocarbons (r = 0.95; *p* < 0.05 for both) and phenolics (r = 0.98 and r = 0.95; *p* < 0.05, respectively). The antimicrobial activity of *Tunisian Punica granatum* L. flower has been linked to their volatile compounds. *Aloe vera* flowers presented some of these compounds, and it is likely that these compounds provide the antimicrobial properties of *Aloe vera* flowers. Fatty acids were detected by SPME technique; however, the data obtained are relative values, and fatty acids have been quantified by GC in Section 3.3. Some of the fatty acids detected by GC have been detected by SPME, and additionally unsaturated fatty acids were only detected by GC.

The volatile profile could be influenced by the different metabolic pathway as the amino acid metabolism. Flower volatiles are used in plant defense or to attract pollinating insects, therefore the qualitative and quantitative profile of volatiles changes in response to flower age, pollination status, endogenous rhythm, and environmental conditions [54].

### 3.3. Fatty Acid Profile

The saturated and polyunsaturated fatty acids (PUFAs) content at the three stages of development of *Aloe vera* flowers are shown in Table 3 and Table 4, respectively. Regarding the qualitative profile, flowers in stage I showed more PUFAs that were not detected in the maturity stage III (Table 3). A total of 13 fatty acids were identified in the stage I and II, whilst in stage III, only 11 fatty acids in the stage III. On the other hand, in terms of the quantitative profile, the highest fatty acids content (saturated and PUFAs) was measured in flowers in the maturity stage II, while the lowest content was observed in the maturity stage III. The main saturated fatty acid is palmitic acid, followed in abundance by stearic acid, caprylic acid, capric acid and arachidic acid. The main PUFAs were linoleic and α-linolenic acids. These results are in line with the data reported in *Aloe arborescence* leaf juice with α-linolenic acid (35%), linoleic acid (27%), and palmitic acid (18%) as the principal fatty acids [2]. The same PUFAs were described as the major fatty acids presented in *Aloe vera* gel [55]. However, López-Cervantes et al. [14] reported eight fatty acids in aloe flowers, with myristoleic acid being the most abundant, followed by palmitic acid, oleic acid, heptadecanoic acid, and stearic acid. These authors did not identify seven fatty acids that we found in flowers: linoleic, α-linolenic, elaicid, 11-eicosenoic, lignoceric, lauric, and myristic acids. However, we did not find myristoleic, heptadecanoic, and erucic acids. The quality and quantity of the fatty acids content is highly dependent on the genotype, geographical area, climatic conditions of the growing season, and drying conditions. López-Cervantes et al. [14] dried the flowers in the sunlight, and possibly the fatty acids—mainly the unsaturated ones—could have degraded due to the high temperature.

α-Linolenic acid is an essential PUFAs omega-3 fatty acid and an important structural component of cell membranes that affects the cell membrane properties and has been linked to cardioprotective properties and also with beneficial properties for diabetes and even cancer [56,57]. Omega-3 fatty acids are also a promising adjuvant therapy for depressive disorder in adults [58]. Another important point to underscore is the optimal balance between omega-3 and omega-6 (linoleic acid) in *Aloe vera* flowers (0.8:1). Further emphasis has been placed on the changes in our dietary habits over the last years in which the consumption of omega-3 in the form of fish, grains, and vegetables has been replaced by the omega-6 fatty acids from cereal oils. These dietary modifications led to the change in the omega-3/omega-6 fatty acid ratio in the diet from 1:1 to 1:15–20. This fact has been especially related to an increase in the rates of depression in recent decades [59]; actually, higher intakes of omega-3 PUFAs, but not omega-6 PUFAs, was associated with lower metabolic diseases [60].

As with our results, the flowers of *Moringa oleifera* presented palmitic acid, linolenic acid, linoleic acid, and oleic acid as the major fatty acids in flowers [61]. Regarding 11-eicosenoic acid (also called gondoic acid), it is found in diverse plant oils such as jojoba, nuts, and extract of *Moringa oleifera* leaves. Lee et al. [62] reported that *M. oleifera* extract contained 54% of cis-11-eicosenoic acid and suggested that 11-eicosenoic acid prevented *Staphylococcus aureus* biofilm formation and could reduce its virulence by diminishing its hemolytic activity. Other saturated fatty acids with biological properties include palmitic acid, with antioxidant, pesticide, and antimicrobial properties; stearic acid, with antibacterial action, as a lubricant and for use in cosmetics; and myristic acid, with antioxidant and anticancer properties [63]. Considering the *Aloe vera* flower’s profile in fatty acids, it could be considered a very interesting byproduct for food, pharmacological, and cosmetic uses.

### 3.4. Carotenoids and RAE

**Figure 3:**
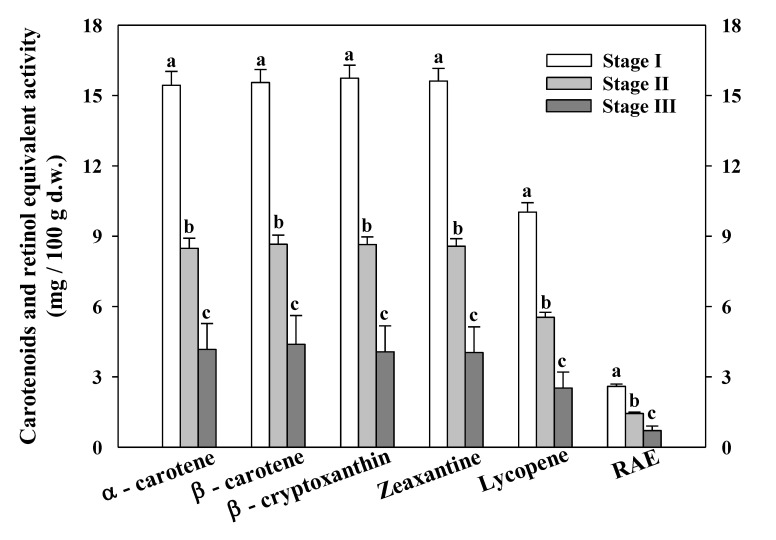
Carotenoids content and retinol activity equivalent (RAE) at different maturity stages of *Aloe vera* flowers. Stage I: immature; Stage II: mature; Stage III: mature, with flowers buds opened (mean ± SD, *n* = 3). ^a–c^ Different letters show significant differences (*p* < 0.05) among the maturity stages.

The individual carotenoids (α-carotene, β-carotene, β-cryptoxanthin, zeaxanthin, and lycopene) content and the RAE were determined in the three maturity stages of *Aloe vera* flowers (Figure 3). 

Both the content of total carotenoids and the RAE decreased with the development of the flowers. Other authors have reported an increase in the carotenoid concentration from young buds to the half open daily flowers [64]. However, carotenoids are synthesized in all types of differentiated plastids but accumulate in high levels in the chloroplasts of green tissues and the chromoplasts of roots, fruits, and flower petals [65]. Among the different carotenoids determined, lycopene was the carotenoid that presented the lowest content in the three maturity stages of *Aloe vera* flowers. López-Cervantes et al. [14] reported the carotenoids content in *Aloe vera* flowers from two different regions, obtaining levels of β-carotene about 1.58–3.78 mg/100 g d.w. depending on the solvent and agitation methods. However, our *Aloe vera* flowers presented an average of 4.39–15.55 mg/100 g d.w. depending on the maturity state. The total carotenoids content in *Aloe vera* flowers (State I) was highest (72.36 mg/100 g d.w.) with the richest content described in *Aloe vera* (*Aloe barbadensis* Miller) leaves (12 mg/100 g f.w.) from the first harvest and irrigated with 25% of its water requirements [5]. However, Walid et al. [66] obtained only 0.021 mg/100 g d.w. in the characterization of *Aloe vera* gel. Kumar and Vallikannan [67] described the carotenoids content in some medicinal plants such as *Aloe vera*, obtaining 0.87 mg/100 g d.w. for α-carotene and 6.59 mg/100 g d.w. for β-carotene. Those researchers found that some green leafy vegetables (*Chenopodium album*, *Amaranthus spinosus*, *Commelina benghalensis*, *Colocasia anti-quorum*, and *Amaranth* sp. (keerai)) contained between 100 mg to 120 mg/100 g d.w. of β-carotene and the medicinal plants, such as *Bacopa monnieri*, *Ocimum sanctum* and *Clitoria ternatea* with 34 mg to 30 mg/100 g d.w. were rich sources of this carotenoid. Therefore, *Aloe vera* flowers can be considered an important source of carotenoids as the flowers showed an intermediate carotenoids value which was between the green leafy vegetables and the medicinal plants. Carotenoids are potent antioxidants and regulate the pathogenesis of some chronic degenerative diseases. β-carotene plays a role in delaying photo-aging induced by UVA, and it can be used for developing skin care products [68]. In addition, products rich in β-carotene may offer one of the successful approaches for improving vitamin A status. Vitamin A deficiency is the leading cause of preventable blindness in children and increases the risk of disease and death from severe infections. This is a public health problem in more than half of all countries, above all in Africa and Southeast Asia [69]. Some of these areas have the climate conditions to growth *Aloe vera*, and consumption of flowers could help to reduce those health problems.

### 3.5. Total Vitamin C

**Figure 4:**
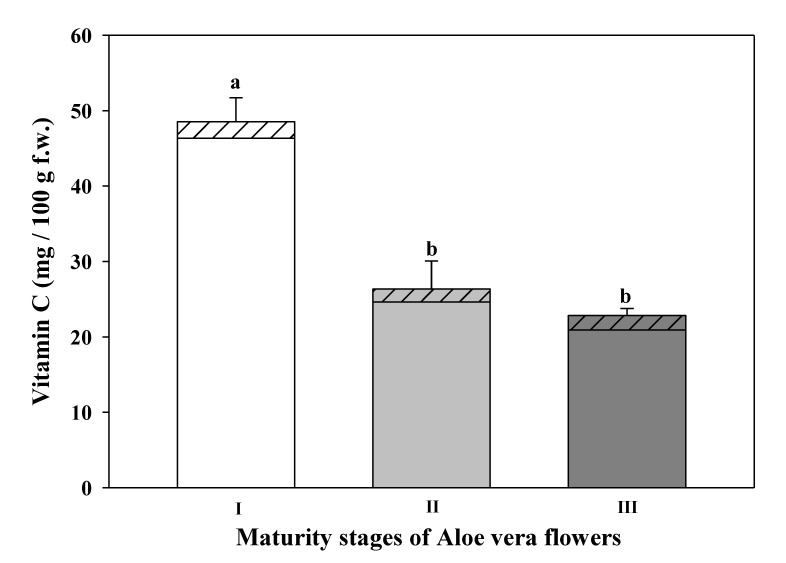
Vitamin C content (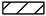 DHAA- dehydroascorbic acid and 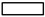 AA-ascorbic acid) at different maturity stages of *Aloe vera* flowers. Stage I: immature; stage II: mature; stage III: mature, with flowers buds opened (mean ± SD, n = 3). ^a-b^ Different letters show significant differences in ascorbic acid (*p* < 0.05) among the maturity stages.

The total vitamin C was determined as the sum of AA and DHAA (Figure 4).

AA was the main form of vitamin C in *Aloe vera* flowers whilst DHAA represented a low percentage of said vitamin C content (around 4.5% to 6.4%). No significant differences were observed in the content of DHAA among the three maturities stages studied of *Aloe vera* flowers. However, the immature stage (I) showed the highest AA content (46.35 ± 3.2 mg/100 g f.w.), although no significant differences were detected between the maturity stages II (24.65 ± 3.74 mg/100 g f.w.) and III (21.62 ± 1.36 mg/100g f.w.). The effect of the development stage in the AA content has been previously described in chrysanthemum petals; the highest ascorbic acid content was found in the just opening period and decreased with its development [70]. However, in daylily flowers and in rose flowers the opposite trend was observed [64]. In relation to the total vitamin C content, the results were similar to those obtained in AA content, due to the low percentage of DHAA present in the samples. The *Aloe vera* flowers showed the highest vitamin C content in the immature stage (I) (48.52 ± 3.2 mg/100 g f.w.), but no significant differences were observed between the stages II (26.34 ± 3.73 mg/100 g f.w.) and III (22.82 ± 0.9 mg/100 g f.w.). This level was close to the total ascorbic acid content measured in a fruit rich in vitamin C: untreated orange juice, which ranged from 50 mg to 60 mg/100 mL f.w. [71]. The vitamin C in *Aloe vera* flowers was about six to three times more than the content measured in *Aloe vera* gel (44.68 ± 2.15 mg/100 g d.w.) reported by Walid et al. [66]. The benefits of vitamin C have been widely reported, with this vitamin being linked to a diminished risk of chronic diseases such as cardiovascular disease, cancer, and cataracts, due to its antioxidant properties. From a cosmetic point of view, skin contains high concentrations of vitamin C, supporting important functions such as stimulating the synthesis of collagen and assisting in UV-induced photodamage protection [72].

### 3.6. Total Phenolic Content

**Figure 5:**
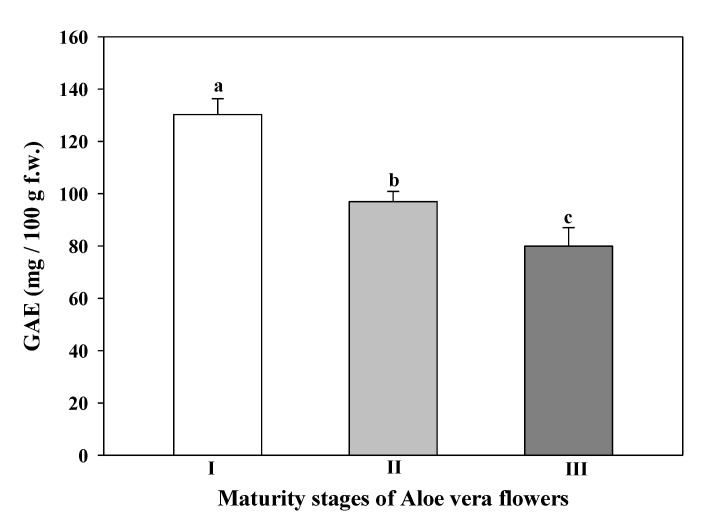
Total phenolic compounds of different stages of maturity of *Aloe vera* flowers. Stage I: immature; stage II: mature; stage III: mature, with flowers buds opened (mean ± SD, n = 3). ^a–c^ Different letters show significant differences (*p* < 0.05) among the maturity stages.

TPC showed a significant decrease with the development of the *Aloe vera* flowers (Figure 5).

The maturity stage I provided the flowers with the highest TPC value (130.24 ± 6.07 mg of GAE/100 g f.w.), and the maturity stage III showed the lowest value (79.95 ± 7.10 mg GAE/100 g f.w.). Debnath et al. [10] described much lower TPC in A*loe barbadensis* flower extracts, 17.52 mg GAE/100 g d.w. on flower ethanol extracts than the present research work. These authors used dried flowers, which could lead to a loss in bioactive compounds depending on the drying method used. Cardarelli et al. [73] studied the phenolic content of leaf exudates from 18 Aloe species. For them, the TPC ranged from 130 mg to 1435 mg GAE/100 g f.w in the exudates of *Aloe vera* leaves being species-dependent, whith *Aloe barbarensis* showing around 320 mg GAE/100 g f.w. Other authors have reported different values of TPC in the *Aloe barbadensis* plant. Lucini et al. [74], obtained a TPC in whole leaf of 1206 ± 60 mg GAE/100 g f.w. and 217 ± 9 mg GAE/100 g f.w. in inner parenchyma, while Chacón et al. [7] reported a TPC in a commercial extract of lyophilized inner *Aloe vera* gel of 114.03 mg GAE/100 g, and Fan et al. [75] described 157 mg GAE/100 g on aloe fresh leaves. These last values are in the range of TPC of flowers of *Aloe barbadensis* observed in our plant material. Polyphenols work as free radical terminators or primary antioxidants; they have a huge impact on health by protecting against some of the reactive oxygen species related chronic diseases as they can scavenge the excess free radicals [1]. The content of TPC in *Aloe vera* flowers could provide rich fresh or dry extract, which is very useful for food, pharmaceutical, or cosmetic uses.

### 3.7. Total Antioxidant Capacity

**Figure 6:**
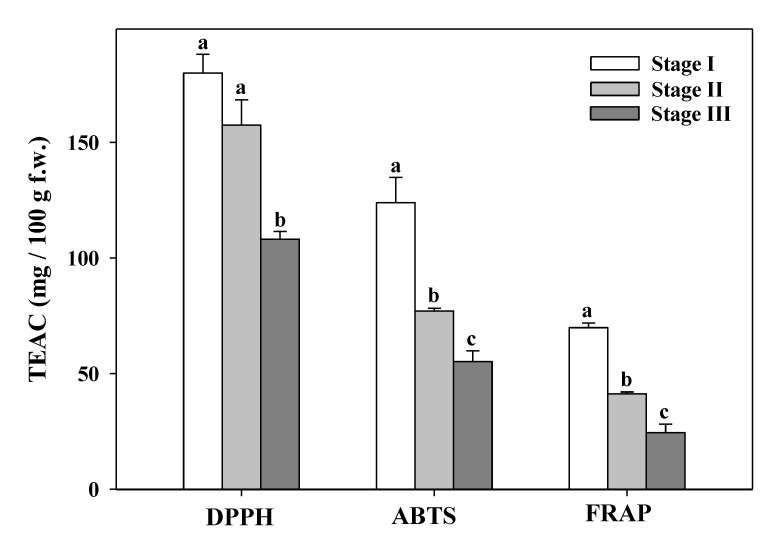
Antioxidant capacity evaluated by three different assays (DPPH, ABTS, and FRAP) at different maturity stages of *Aloe vera* flowers. Stage I: immature; stage II: mature; stage III: mature, with flowers buds opened (mean ± SD, n = 3). ^a–c^ Different letters show significant differences (*p* < 0.05) among the maturity stages.

The total antioxidant capacity evaluated by three different assays (DPPH, ABTS, and FRAP) provided the same trend in the three-flower maturity state; the highest and lowest values were obtained in the maturity stage I and maturity stage III, respectively (Figure 6).

This same behavior was found in the content of carotenoids, ascorbic acid, and TPC. The highest antioxidant capacity was obtained by DPPH assay (Figure 6). The antioxidant activity of tea flowers (with 5 maturity developmental stages) increased to reach a maximum at stage 3 then decreased in half open flower and full bloom [30]. In our case, *Aloe vera* flowers in stage I showed a high antioxidant capacity (179.91 ± 8.16 mg TEAC/100 g f.w.) decreasing with flowers buds open, higher than the antioxidant capacity measured by DPPH in freeze-dried *Aloe vera* (*Aloe ferox*) leaves (890.77 mg TEAC/100 g d.w.) by Corpus-González et al. [76]. Vega-Gálvez et al. [77] obtained the highest antioxidant activity (DPPH) in *Aloe vera* gel summited under high hydrostatic pressure (300 MPa) after 35 days of storage (0.655 mg/100 mL f.w.). Cardarelli et al. [73] suggested that phenolic compounds present in leaf exudates provided most of the antioxidant capacity as reported by other researchers in a varied range of herbs, vegetables, teas, and fruit [78]. Additionally, ethanolic and aqueous extracts of the bagasse (byproducts of *Aloe vera* processing leaves) have shown antioxidant and antifungal activities in industrial crops during pre- and post-harvest stages [37]. *Aloe vera* leaf is known for its properties such as minimizing the severity of cancer, antioxidant activity, diabetes, inflammation, and microbial diseases, among others [79]. Therefore, the antioxidant properties of *Aloe vera* flowers could have an important application in the food industry, thus providing an income for farmers if the flowers are considered as a byproduct rather than as a residue. In fact, natural products with antioxidant properties have been extensively utilized as health-promoting herbal products as well as natural additives in the food industry. Grajzer et al. [80] considered rose hip oils higher than 62.6 mg TEAC/100 g oil to offer an important antioxidant activity, therefore the values measured in *Aloe vera* flowers by the three antioxidant assays (DPPH, FRAP, and ABTS) have showed a good level.

## 4. Conclusions

*Aloe vera* flowers exhibited significant vitamin C, TPC, and carotenoid content, with a high antioxidant capacity. The content in citric and malic acids and trigonelline was also notable. Additionally, *Aloe vera* flowers presented a considerable amount of fatty acids; the major concentration was identified in PUFAs such as α-linolenic acid and linoleic acid, with an optimal balance between them. The main saturated fatty acids were capric acid, palmitic acid, stearic acid, caprylic acid and arachidic acid. VOCs profile indicated benzyl alcohol as one of the main volatile compounds. The different stages of maturity exhibited significant qualitative and quantitative differences in the bioactive compounds studied. The maturity stage I showed the highest content in practically all the analyzed compounds, while the maturity stage II showed the highest fatty acid content.

This study demonstrates that *Aloe vera* flowers are a byproduct with a valuable source of active ingredients, to which have been attributed numerous health properties with potential applications in the cosmetic, pharmaceutical, nutraceutical and food industries. Moreover, depending on the compound of interest, it could be worthwhile harvesting flowers at maturity stage I, thereby reducing the energy consumption of flowers from the plant, favoring plant development. Further studies will be necessary to corroborate the in vitro and in vivo *Aloe vera* flowers properties and their potential for other products, albeit from freeze-dried flowers or by the extraction of bioactive compounds (tinctures, hydroalcoholic and glycolic extracts, etc.).

## Figures and Tables

**Table 1:** Primary metabolites (mg/g d.w.) of the different maturity stages of *Aloe vera* flowers. Stage I: immature; stage II: mature; stage III: mature with stamens exposed to the outside.

	Stage I	Stage II	Stage III
**Amino acids**			
Glutamine	0.613 ± 0.027 ^a^	0.748 ± 0.076 ^a^	0.726 ± 0.186 ^a^
Aspartate	0.273 ± 0.029 ^a^	0.193 ± 0.027 ^b^	0.252 ± 0.042 ^ab^
Alanine	0.169 ± 0.027 ^a^	0.206 ± 0.030 ^a^	0.201 ± 0.013 ^a^
Phenylalanine	0.263 ± 0.020 ^a^	0.135 ± 0.029 ^b^	0.111 ± 0.011 ^b^
GABA	0.168 ± 0.032 ^a^	0.119 ± 0.018 ^a^	0.122 ± 0.022 ^a^
Threonine	0.119 ± 0.023 ^a^	0.091 ± 0.017 ^a^	0.109 ± 0.036 ^a^
Tyrosine	0.185 ± 0.013 ^a^	0.151 ± 0.005 ^b^	0.138 ± 0.025 ^b^
Valine	0.096 ± 0.021 ^a^	0.074 ± 0.018 ^a^	0.087 ± 0.007 ^a^
Isoleucine	0.064 ± 0.022 ^a^	0.047 ± 0.014 ^a^	0.064 ± 0.003 ^a^
**Free sugars Composition**			
Fructose	8.14 ± 0.25 ^c^	11.81 ± 0.16 ^b^	28.57 ± 0.88 ^a^
Glucose	7.57 ± 0.53 ^c^	17.34 ± 0.74 ^b^	42.78 ± 7.24 ^a^
Sucrose	11.07 ± 0.92 ^a^	9.78 ± 0.19 ^a^	3.12 ± 0.83 ^b^
Trehalose	0.10 ± 0.00 ^c^	0.14 ± 0.03 ^b^	0.34 ± 0.02 ^a^
**Organic acids**			
Citric Acid	4.315 ± 0.216 ^a^	2.919 ± 0.223 ^b^	1.552 ± 0.097 ^c^
Malic acid	2.134 ± 0.163 ^ab^	2.404 ± 0.183 ^a^	2.084 ± 0.087 ^b^
Acetic acid	0.023 ± 0.002 ^b^	0.026 ± 0.001 ^b^	0.044 ± 0.013 ^a^
Formic acid	0.009 ± 0.000 ^c^	0.010 ± 0.000 ^b^	0.015 ± 0.001 ^a^
Fumaric acid	0.014 ± 0.001 ^a^	0.013 ± 0.001 ^a^	0.012 ± 0.001 ^a^
**Nucleoside derivatives**			
AMP	0.140 ± 0.016 ^a^	0.131 ± 0.003 ^ab^	0.116 ± 0.009 ^b^
Adenosine	0.105 ± 0.014 ^a^	0.096 ± 0.005 ^a^	0.087 ± 0.005 ^a^
**Other metabolites**			
Choline	0.492 ± 0.024 ^a^	0.458 ± 0.020 ^ab^	0.426 ± 0.028 ^b^
Trigonelline	0.519 ± 0.014 ^a^	0.328 ± 0.004 ^b^	0.180 ± 0.014 ^c^
Ethanol	0.025 ± 0.004 ^a^	0.019 ± 0.003 ^b^	0.013 ± 0.001 ^c^

Means (*n* = 3 ± SD). ^a–c^ Different letters in the same row indicate significant differences (*p* < 0.05) for maturity stages. GABA: Gamma γ-aminobutyric acid.

**Table 2:** List of compounds with quality higher than 80 with percentage contribution to total area of all peaks of volatiles compounds of the *Aloe vera* flowers at three maturity stages. Stage I: immature; stage II: mature; stage III: mature with stamens exposed to the outside.

Compound	CAS Number	RT (min)	Stage I (% Relative)	Stage II (% Relative)	Stage III (% Relative)
**VOCs**					
1-Pentanal	110-62-3	1.650	0.66	0.55	0.66
Butanoic acid	107-92-6	2.251	1.67	1.34	1.85
1-Hexanal	66-25-1	2.488	1.82	1.75	2.08
2-Hexenaldehyde	6728-26-3	3.185	6.79	4.64	5.37
Benzene ethenyl	629-20-9	3.786	2.61	1.89	2.02
1-heptanal	111-71-7	3.980	1.35	1.28	-
Benzaldehyde	100-52-7	5.198	4.94	9.27	5.00
Hexanoic acid ethyl ester	123-66-0	6.090	0.75	-	-
Benzyl alcohol	100-51-6	6.860	23.88	16.99	27.52
Benzeneacetaldehyde	122-78-1	7.109	1.81	1.48	1.67 *
Acetophenone	98-86-2	7.621	1.35	0.62 *	-
Phenol, 3-methyl	108-39-4	7.807	0.71	0.69	0.65
Formic acid phenylmethyl ester	104-57-4	7.854	0.93	0.77 *	1.21
1-Nonanal	124-19-6	8.370	5.96	5.66	5.98
Acetic acid benzyl ester	140-11-4	9.731	0.75 *	-	0.75
Dodecane	112-40-3	10.458	0.75 *	0.57	0.53 *
Tetradecane	629-59-4	15.237	1.67	2.93	1.60
**Fatty acids and fatty acid esters**					
Caproic acid	142-62-1	5.676	8.12	6.35	7.32
Octanoic acid methyl ester	111-11-5	8.831	0.83	-	0.55
Caprylic acid	124-07-2	9.900	4.39	3.04	4.40
Decanoic acid methyl ester	110-42-9	13.44	0.64 *	0.90	0.72 *
Capric acid	334-48-5	14.442	1.34	1.00	1.23
Dodecanoic acid methyl ester	111-82-0	18.269	3.75	4.95	3.63
Lauric acid	143-07-7	19.186	5.98	8.06	7.71
Tetradecanoic acid methyl ester	124-10-7	22.899	-	0.55	-
Myristic acid	544-63-8	23.652	-	0.70	-

* compound with quality lower than 80. Symbol - means not identified compound. CAS: Chemical Abstracts Service. RT: Retention Time. VOCs: Volatile organic compounds.

**Table 3:** Saturated fatty acids (mg/100 g d.w.) of the three maturity stages of *Aloe vera* flowers. Stage I: immature; stage II: mature; stage III: mature with stamens exposed to the outside.

Maturity Stage	Caprylic acid (C8:0)	Capric acid (C10:0)	Lauric acid (C12:0)	Myristic acid (C14:0)	Palmitic acid (C16:0)	Stearic acid (C18:0)	Arachidic acid (C20:0)	Lignoceric acid (C24:0)
**I**	59.5 ± 9.5 ^a^	51.9 ± 4.1 ^a^	26.0 ± 7.0 ^a^	1.5 ± 0.3 ^ab^	436.2 ± 7.3 ^a^	105.2 ± 5.4 ^b^	27.2 ± 7.1 ^a^	11.9 ± 1.4 ^b^
**II**	62.9 ± 12.3 ^a^	60.9 ± 3.9 ^a^	28.4 ± 5.5 ^a^	2.1 ± 0.2 ^a^	414.9 ± 98.1 ^a^	167.8 ± 22.9 ^a^	44.4 ± 5.4 ^a^	27.4 ± 8.9 ^a^
**III**	53.8 ± 4.7 ^a^	51.4 ± 8.4 ^a^	16.6 ± 3.8 ^a^	0.7 ± 0.2 ^b^	189.0 ± 30.6 ^b^	110.0 ± 5.7 ^b^	38.8 ± 5.7 ^a^	-

Means (*n* = 3 ± SD). ^a,b^ Different letters in the same column indicate significant differences (*p* < 0.05) for maturity stages.

**Table 4:** Polyunsaturated unsaturated fatty acids (mg/100 g d.w.) of the three maturity stages of *Aloe vera* flowers. Stage I: immature; stage II: mature; stage III: mature with stamens exposed to the outside.

Maturity Stage	Elaidic acid (C18:1n9)	Oleic acid (C18:1n9)	Linoleic acid (C18:2n6)	α-Linolenic acid (C18:3n3)	11-Eicosenoic acid (C20:1n9)
**I**	176.4 ± 35.4 ^a^	85.1 ± 5.4 ^a^	826.4 ± 22.8 ^a^	609.3 ± 8.8 ^a^	16.4 ± 3.2 ^b^
**II**	165.1 ± 26.6 ^a^	94.7 ± 12.0 ^a^	802.5 ± 108.3 ^a^	785.8 ± 139.0 ^a^	62.0 ± 3.0 ^a^
**III**	73.4 ± 20.9 ^b^	6.5 ± 5.0 ^b^	325.1 ± 46.5 ^b^	270.6 ± 55.6 ^b^	-

Means (*n* = 3 ± SD). ^a,b^ Different letters in the same column indicate significant differences (*p* < 0.05) for maturity stages. Symbol - means not identified compound.
